# The Public Health Services: Their Attraction for Practitioners

**Published:** 1917-09-08

**Authors:** 


### THE PUBLIC HEALTH SERVICES.

### Their Attraction for Practitioners.

This country has now passed through the third year of a war unprecedented and beyond imagination according to all previously conceived standards. It would be no matter for shame if, during such a year, the Public Health Services had perforce curtailed, or even altogether dropped, much routine sanitary work. The national steadiness and adaptability under trial have been truly remarkable. In the care of the public health not only has supervision been maintained with but little modification, but there has been sustained activity and even actual progress. This progress has been especially shown in the practical working of the Venereal Diseases Regulations, in the activity and enthusiasm thrown into schemes for maternity and infant welfare, and in the proposal to establish a Ministry of Health. The arrangements made for the treatment of venereal diseases at the cost of the State are, like those made for the treatment of tuberculosis under the National Insurance Act, an exemplification of the principle that preventive and curative medicine are in practice inseparable.

It has long been accepted that the State cannot deal effectively with infectious disease without offering treatment to the infected individual. The campaign which has aimed at the prevention of venereal disease, and of which the official regulations are largely the outcome, sought also the adoption of repressive measures of a different character. We have referred before to the fact that public health developments and legislation have shown a definite trend towards the supervision and protection of the mother, the infant, and the child. Pleas of national needs and responsibilities have been strengthened by the war and have been pressed during the past summer with an energy which reached its climax in the much-advertised Baby Week of July. Opinions vary upon the merits and values of baby-shows, but there can be no doubt that the publicity and interest aroused by Baby Week are bearing fruit and will prove a useful and enduring stimulus to infant clinics, mothers' welcomes, and *crèches*. The matter of provision for care during childbirth, involving as it does supervision of the distribution and employment of midwives, has also been brought under consideration.

The proposal to create a Ministry of Health, brought forward unexpectedly, and for a time somewhat feverishly discussed, has been necessarily dropped or, perhaps it should rather be said, shelved. It is a proposal which must be renewed at a later, and more propitious, date. The fields of medicine and hygiene are now too vast and important for State medicine to be allowed to drift further, in isolated sections, into the hands of multiple authorities and to become yet more unwieldy. The great and increasing drain upon public funds constitutes in itself a strong argument in favour of the formation of a single public health governing department; still more important is the need, now becoming imperative, for organisation and co-ordination of the various medical branches of public assistance. We need not here enlarge upon existing anomalies, upon the overlapping and the waste of our multiple "authorities," or upon the jealousies which the mere suggestion of a Ministry of Health has shown to exist. Foremost amongst the needed changes in administration still stands Poor-Law reform.

The Public Health (including the Poor-Law) Service is deservedly attractive to medical men and women, to an increasing number of whom it offers employment sufficiently full of promise, variety, and interest. Appointments in the Service are, generally speaking, and with the exception of the higher administrative posts, not highly paid, and they only rarely offer any prospect of pension or any security of tenure, but their prospects are steadily improving and are already sufficiently good to attract applicants of a high professional standard. Whilst experience in general practice is valuable as a prelude to public health medical work, the special training required for this work is long and arduous, and it is generally necessary for the graduate to proceed, shortly after "qualifying," to obtain a diploma in public health or sanitary science. Meanwhile the training and experience gained by holding a resident appointment in a general, and also if possible in a fever, hospital is invaluable, as is also practice in bacteriological work.

We need not set out at length the requirements of study for a public health diploma, which is registrable, and the possession of which is a *sine quâ non* for most appointments in the Service. The examinations are of wide scope, and preparation entails prolonged study and no little expense of time and money, the General Medical Council having taken steps to ensure the possession by candidates of a "distinctly high proficiency, scientific and practical, in all the branches of study which concern the public health." Candidates are required by some examining bodies to have been in possession of a registrable medical qualification for at least twelve months before entry for the examination and to have attained a specified age. The knowledge required is fascinating and absorbing in its interest, and it can safely be asserted that no student who can devote to it the necessary time and application will ever afterwards regard these as having been other than well spent.

